# Hemodynamic imaging parameters in brain metastases patients – Agreement between multi-delay ASL and hypercapnic BOLD

**DOI:** 10.1177/0271678X231196989

**Published:** 2023-08-26

**Authors:** Eva E van Grinsven, Jamila Guichelaar, Marielle EP Philippens, Jeroen CW Siero, Alex A Bhogal

**Affiliations:** 1 Department of Neurology & Neurosurgery, University Medical Center Utrecht Brain Center, Utrecht University, Utrecht, The Netherlands; 2 Department of Radiation Oncology, University Medical Center Utrecht, Utrecht, The Netherlands; 3 Department of Radiology, University Medical Center Utrecht, Utrecht, The Netherlands; 4 Spinoza Center for Neuroimaging, Amsterdam, Netherlands

**Keywords:** Brain metastases, cerebral blood flow, cerebrovascular reactivity, hemodynamics, hemodynamic lag

## Abstract

Arterial spin labeling (ASL) MRI is a routine clinical imaging technique that provides quantitative cerebral blood flow (CBF) information. A related technique is blood oxygenation level-dependent (BOLD) MRI during hypercapnia, which can assess cerebrovascular reactivity (CVR). ASL is weighted towards arteries, whereas BOLD is weighted towards veins. Their associated parameters in heterogeneous tissue types or under different hemodynamic conditions remains unclear. Baseline multi-delay ASL MRI and BOLD MRI during hypercapnia were performed in fourteen patients with brain metastases. In the ROI analysis, the CBF and CVR values were positively correlated in regions showing sufficient reserve capacity (i.e. non-steal regions, *r_rm_* = 0.792). Additionally, longer hemodynamic lag times were related to lower baseline CBF (*r_rm_* = −0.822) and longer arterial arrival time (AAT; *r_rm_* = 0.712). In contrast, in regions exhibiting vascular steal an inverse relationship was found with higher baseline CBF related to more negative CVR (*r_rm_* = −0.273). These associations were confirmed in voxelwise analyses. The relationship between CBF, AAT and CVR measures seems to be dependent on the vascular status of the underlying tissue. Healthy tissue relationships do not hold in tissues experiencing impaired or exhausted autoregulation. CVR metrics can possibly identify at-risk areas before perfusion deficiencies become visible on ASL MRI, specifically within vascular steal regions.

## Introduction

Advances in imaging techniques have made MRI a powerful tool for investigating not only brain function but also brain physiology and auto-regulatory status. One such method that now sees routine clinical application is Arterial Spin Labeling (ASL). ASL is non-invasive, and can provide diagnostically relevant, quantitative parameters related to hypo- or hyper-perfusion.^1–3^ Multi post label delay (multi-PLD) methods can even be used to infer the presence of collateral cerebral blood flow (CBF) pathways through their ability to estimate arterial arrival time (AAT) or characterize arterial transit artifacts (ATA). Together these ASL metrics can provide valuable physiological information in healthy subjects and can also identify disease-related auto-regulatory changes in patient populations.

A major cerebral auto-regulatory mechanism responsible for maintaining adequate CBF is cerebrovascular reactivity (CVR). CVR is reflective of smooth-muscle cell mediated blood flow control, and represents a major compensatory mechanism in diseases that compromise cerebral hemodynamics.^4–6^ The CVR response can be assessed and spatially mapped using Blood oxygenation level-dependent (BOLD) MRI in combination with controlled hypercapnic stimuli. This technique is distinct from resting-state and/or task-based BOLD MRI, where either spontaneous or evoked *neuronal* signals modulate the BOLD signal change, providing information related to brain function. CVR measurements can be interpreted as regional indicators of healthy or disease-impaired vasculature.^7^ Impairments often manifest as negative signal responses, where a vasodilatory stimulus can cause paradoxical decrease in CBF (known as vascular steal) to an area with exhausted dilatory reserve, due to a reduction in vascular resistance in neighboring, non-exhausted regions.^8^ Finally, analysis of dynamic CVR characteristics can provide information on temporal aspects of the CVR response that are encompassed in the hemodynamic response lag^9–11^

Changes in perfusion characteristics and cerebrovascular function have been found in multiple different patient populations, including patients with brain tumors.^12–14^ Growth of intracranial masses, like primary brain tumors or brain metastases, can cause local disruptions of the hemodynamic environment. Metastatic cells have to proceed through a range of developmental steps in order to form macrometastases; (1) cells arrest in the small microvessels, (2) cells extravasate to enter the brain tissue, (3) cells perpetuate into a strict perivascular position, and (4) vascularization is secured through either co-optive or angiogenic growth depending on the tumor type.^15^ By vascular co-option, angiogenesis and dilation of blood vessels associated with brain metastases, the macrometastases subsequently ensure adequate blood supply and thus alter the vascular microenvironment.^16–18^ The vasogenic edema often surrounding these brain metastases, caused by the local blood-brain barrier disruption^19^, can lead to additional physiological changes. All these brain metastases related physiological changes are likely to be reflected in hemodynamic MRI measurements. ASL and CVR measure associated responses, both describing the status of the vascular network. Thereby they can provide complementary information, however, it remains unclear how ASL and CVR parameters relate in different tissue types or under different hemodynamic circumstances.

In the current exploratory study we test how ASL parameters measured during a physiological steady state relate to functional vascular parameters as measured using CVR in a population of patients with brain metastases. The expected tissue-heterogeneity caused by the brain metastases growth provides a testing ground to answer this question. CBF and AAT were compared to CVR magnitude and hemodynamic lag using two different approaches. Firstly, the inter-modality agreement between ASL and CVR was tested by comparing group values for different tissue types (grey matter (GM), white matter (WM), edema and brain metastases) for both ASL and CVR metrics. Secondly, we assessed the spatial correlation between ASL and CVR metrics throughout the brain using both a ROI and voxelwise approach. Hereby a distinction was made between brain areas showing adequate vascular responses and those exhibiting vascular steal phenomena. Understanding how baseline vascular physiology relates to dynamic vascular processes in cases with brain metastases will aid in interpreting these measures in future research.

## Materials and methods

### Study set-up and population

For the current retrospective observational study, MRI datasets were included from the ongoing Assessing and Predicting Radiation Influence on Cognitive Outcome using the cerebrovascular stress Test (APRICOT) study. Participation consists of an elaborate neurocognitive exam and MRI scans, including a BOLD MRI scan during breathing challenges, before radiotherapy and approximately three months after radiotherapy. For the purpose of this study only the MRI data acquired *before* radiotherapy during the period between October 2020 and February 2022 was used. The study population consists of adult patients (≥18 years) with either radiographic and/or histologic proof of metastatic brain disease that were referred to the radiotherapy department of the UMC Utrecht for radiation therapy of the brain metastases. Specific in- and exclusion criteria for participation in the APRICOT study are listed in Supplementary Methods. The study was performed in accordance with the Declaration of Helsinki^20^ and the UMCU institutional ethical review (Medisch-Ethische Toetsingscommissie (METC) NedMec) approved the study (METC# 18-747). Written informed consent was obtained from all participants prior to participation.

### Data acquisition

#### Imaging protocol

The participants were scanned on a 3 Tesla MRI scanner (Achieva, Philips Medical Systems, Best, The Netherlands) using a 32 channel receive coil. Whole-brain multi-slice single shot FE-EPI BOLD images (TR = 1050 ms, TE = 30 ms, flip angle 65°, resolution 2.292 × 2.292 × 2.5 mm^3^, acquisition matrix 96 × 96 × 51, 1000 dynamics, multi-band factor = 3) were acquired throughout a computer controlled hypercapnic breathing protocol (described below). Perfusion data was acquired using a multi-PLD ASL sequence. A whole volume was acquired at each of the 4 post-labeling delays (660, 1325, 1989, 2654 ms), using a pCASL Look-Locker multi-slice EPI read-out (total scan time = 240 s, labelling train duration = 1650 ms, TR = 5 s, TE = 12 ms, flip angle 25°, acquired resolution: 3 × 3 × 7 mm^3^, acquisition matrix: 80 × 80 × 17, 23 averages, SENSE factor = 2, 2 background suppression pulses). A total of 23 label-control pairs were acquired. The first acquired ASL dataset pair here has the labelling, saturation pulses and background suppression turned off and are the M0 images for each post-label delay. An additional dataset pair was acquired for EPI phase (distortion) correction. No breathing challenges were performed during ASL imaging. The ASL was planned using a phase contrast angiography scan, with the labeling plane carefully placed perpendicular to the internal carotid arteries and vertebral arteries. Additionally, a 3 D spoiled gradient echo (SPGR) sequence (TR = 8 ms, TE = 3.25 ms, flip angle 10°, isotropic resolution 1 mm, acquisition matrix 240 × 240 × 180), a 3 D T2-weighted FLAIR sequence (TR 4800 ms, TE 240 ms, TI = 1650 ms, flip angle 90°, isotropic resolution: 1 mm, acquisition matrix 256 × 256 × 182), as well as a SWI (TR 50 ms, flip angle 17°, inplane slice thickness 2 mm, acquisition matrix 384 × 383 × 63) were acquired as an anatomical reference. A minimum-intensity-projection was constructed from the SWI data, using an in-house developed Matlab script (Matlab R2020a, The MathWorks, Inc., Natick, Massachusetts, United States).

#### Clinical CT and MRI acquisition

CT and MRI scans were acquired as part of clinical care as usual 1 to 5 days before receiving radiotherapy. CT scans were acquired on a Brilliance Big bore 22 scanner (Philips Medical Systems, Best, The Netherlands) with a tube potential of 120 kVp, matrix size of 512 × 512 and inplane slice thickness of 1 mm. The participants were scanned on a 1.5 Tesla MRI scanner (Inginia, Philips Medical Systems, Best, The Netherlands) using a 15 channel receive coil. A 3 D SPGR sequence after injection of 0.1 ml gadovist/kg was performed (TR = 7.6 ms, TE = 3.4 ms, flip angle 8°, isotropic resolution: 1 mm, acquisition matrix 232 × 232 × 170). The clinician uses this clinically acquired MRI registered to the CT, to plan the radiotherapy and delineate the so-called gross tumor volume (i.e. brain metastases). The CT and corresponding gross tumor volume were extracted for each patient. For the current analysis, a distinction was made between newly treated brain metastases, resection cavities of brain metastases and previously irradiated brain metastases.

#### Breathing protocol

**Figure 1.**
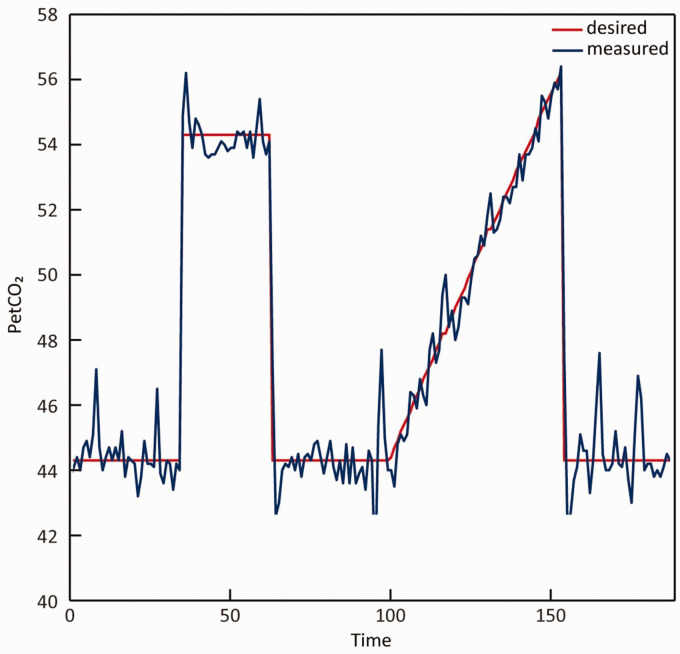
CO_2_ breathing trace of a representative subject during the breathing challenge. The blue line indicates the actual measured PetCO_2_ values, while the red line indicates the desired values based on the protocol. PetCO_2_: end-tidal CO_2_.

Hypercapnic stimuli were delivered using a computer-controlled gas blender and sequential delivery system (RespirAct^TM^, Thornhill Research Institute, Toronto, Canada). The breathing mask was sealed to the patients’ face using transparent dressings (Tegaderm, 3 M, St. Paul, MN, US) to acquire an air tight seal. Before starting the breathing challenges inside the MRI scanner, patients performed a test round with a CO_2_ challenge which is similar to the CO_2_ block given during the protocol. Only after successful completion of the test round, the breathing challenges inside the scanner were performed (see Figure 1). The breathing challenges started with a 5-minute baseline period, followed by a block-shaped increase of end-tidal CO_2_ (PetCO_2_) 10 mmHg relative to a patients’ baseline for 90 seconds. After this so-called CO_2_-block, PetCO_2_ values returned to baseline values for 120 seconds, followed by a PetCO_2_ ramp increase of 12 mmHg relative to patients’ baseline for 180 seconds after which patients returned to baseline for 90 seconds.

### Pre-processing

Pre-processing steps were performed using the Oxford Centre for Functional MRI of the BRAIN (FMRIB) Software Library (FSL – version 6.0; see Supplementary Figure 1).^21^ First, both the T1 and T2FLAIR images were brain extracted using BET.^22^ Tissue segmentation into grey matter (GM), white matter (WM) and cerebrospinal fluid (CSF) was performed on the T1 image using FSL Automated Segmentation Tool (FAST).^23^ Additionally, an edema mask was created based on the T1 and T2FLAIR images using the lesion growth algorithm as implemented in the Lesion Segmentation Tool (LST, https://www.statistical-modelling.de/lst.html) for SPM.^24^ Based on previous experience, the initial threshold was set at 0.14. The resulting edema mask was manually adapted to eliminate any false positives or false negatives from the LST edema mask. The CT image was registered to the T1 image using FMRIB’s Linear Image Registration Tool (FLIRT).^22,25^

BOLD data was motion corrected (MCFLIRT)^22^, corrected for geometric distortion using TOPUP^26,27^ and linear spatial co-registered to the T1 image using the ‘epi_reg’ function.^22,25^ The T1w segmentation masks, edema masks and gross tumor volume masks were translated to the functional (i.e. BOLD) space using the inverse of the matrices output by epi_reg. For the ASL data, the reconstructed T1 (M0) was spatially registered to the pre-TOPUP mean BOLD image. The resulting transformation matrix as well as the distortion-correction fields were then applied to the ASL images in order to move them to functional patient space.

### Data analysis

#### MRI analysis

**Figure 2.**
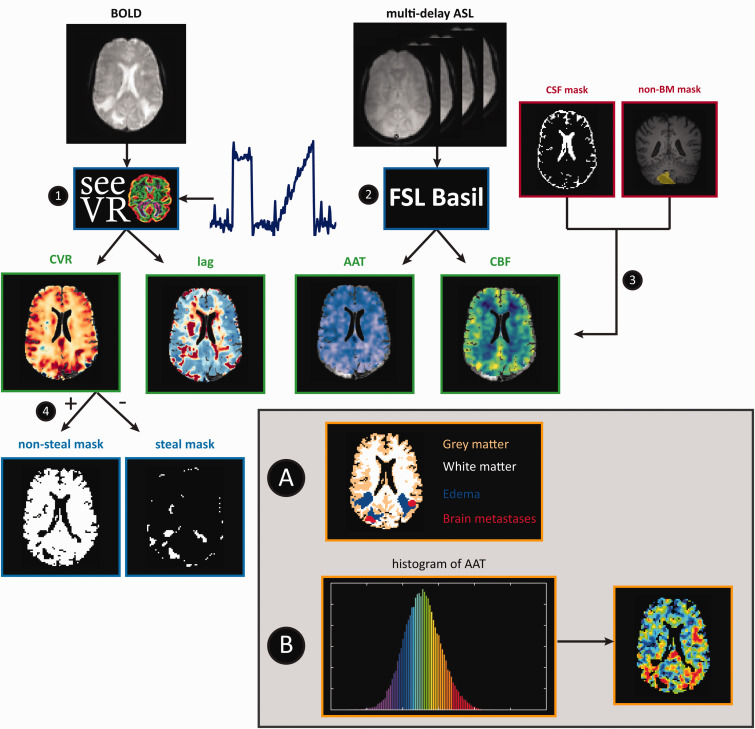
Data analysis steps for the BOLD and ASL MRI data. 1. BOLD time series and PetCO_2_ traces were used to generate CVR and hemodynamic lag maps. 2. Quantitative AAT and CBF maps were generated based on Multi-delay ASL data 3. CSF and previously resected or irradiated brain metastases were excluded from all the maps. 4. Based on the CVR values, the brain was segmented into non-steal and steal regions of interest (ROI). Parameter maps were analyzed comparatively (shown in grey). For analysis A, the brain was divided into GM, WM, edema and brain metastases ROIs for each individual and the mean and standard deviation were calculated for each hemodynamic parameter map (CBF, AAT, CVR and hemodynamic lag) for each tissue-ROI. For analysis B, the AAT data were sorted and the AAT maps were divided into ROIs each containing 5% of the sorted data (‘5%-bins’ - visualized by the colored histogram) for each individual. For each bin-ROI the mean value of each hemodynamic parameter map (CBF, AAT, CVR and hemodynamic lag) was calculated and used in subsequent repeated-measures correlation analysis. AAT: arterial arrival time; ASL: arterial spin labeling; BM: brain metastases; BOLD: blood oxygenation level-dependent; CBF: cerebral blood flow; CSF: cerebrospinal fluid; CVR: cerebrovascular reactivity; GM: grey matter; PetCO_2_: end-tidal CO_2_; ROI: region of interest; WM: white matter.

The pipeline to process and compare the BOLD and ASL data is visually depicted in Figure 2. CVR and hemodynamic lag maps were derived using the open-source seeVR toolbox (available at https://www.seevr.nl/).^28^ In brief, in order to remove signal contribution from large veins that might overshadow tissue responses, a modified whole-brain mask was generated using the ‘remLV.m’ function of the seeVR toolbox.^28^ For this, a temporal noise-to-signal (tNSR) map was calculated by taking the inverse of the temporal signal to noise (tSNR) map. Next, voxels showing values higher than the 98th percentile tNSR value were removed from the original whole brain-mask. This modified mask was then used in subsequent analyses.^28^

Next, a manual bulk alignment was performed between the PetCO_2_ and average GM time-series to minimize alignment errors that can occur when using automated correlation methods. Thereby, any bulk delays between end-tidal gas measurements at the lungs and BOLD signal responses in the brain were accounted for. Residual motion signals with a correlation higher than 0.3 with the GM time-series, along with a linear drift term were regressed out using a general linear model. BOLD data was then temporally de-noised using a wavelet-based approach^29^ and was spatially smoothed using a 3 D Gaussian kernel (FWHM: 4 × 4 × 7 mm^3^). This kernel was chosen in order to best match the effective spatial resolution of the ASL data. BOLD data and corresponding PetCO_2_ traces were interpolated by a factor 4 (effective TR: 262.5 ms) to identify sub-TR signal displacements in subsequent hemodynamic lag analysis. A linear regression was performed between the bulk-aligned PetCO_2_ trace with each processed BOLD voxel time-series. The slope of this linear regression was taken as the CVR (ΔBOLD/mmHg PetCO_2_). Hemodynamic lag maps were generated using the Rapidtide^10,30^ approach as implemented in the seeVR toolbox^28^ and described previously.^31^

The multi-delay ASL was processed using the open-source ClinicalASL toolbox (available at https://github.com/JSIERO/ClinicalASL) and FSL BASIL for quantitative CBF maps.^32^ A T1-weighted image was reconstructed based on the M0 images using the ‘ASLT1fromM0Compute.m’ function. In short, as we used a Look-Locker read-out, the signal evolution over the multiple PLDs will show a T1-weighted signal response that was used to generate a surrogate T1 weighted image. This image had sufficient T1 contrast to be used for image registration (i.e. improved contrast compared to any single M0 or label/control image). Outlier removal was performed based on the standard deviation and tissue variance. Quantitative CBF and AAT maps were generated using the BASIL tool (Figure 2(2)).^32^

#### Statistical analysis

The CSF mask and non-brain metastases mask (containing areas of previously resected or irradiated brain metastases) were excluded from the hemodynamic parameter maps (Figure 2(3)). Thereby, all analyzed tissue consists of either brain matter (GM and WM), edema or non-treated brain metastases. Next, the CVR values were used to divide brain into regions of vascular steal versus no vascular steal. In this study, steal was conceptualized as voxels containing negative CVR values (i.e. regions in which BOLD signal intensity decreased with increase in PetCO_2_; Figure 2(4)).

**Figure 3.**
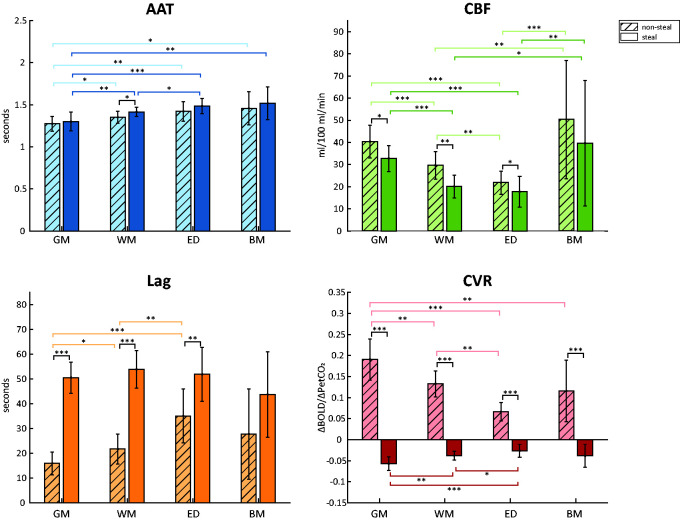
Group mean values per MRI metric (AAT, CBF, hemodynamic lag and CVR) and per tissue type (GM, WM, edema, brain metastases). Error bars represent the standard deviation. Asterisks indicate significant differences between group values with *p < .05, **p < .01, ***p < .001. AAT: arterial arrival time; BM: brain metastases; CBF: cerebral blood flow; CVR: cerebrovascular reactivity; ED: edema; GM: grey matter; WM: white matter.

**Figure 4.**
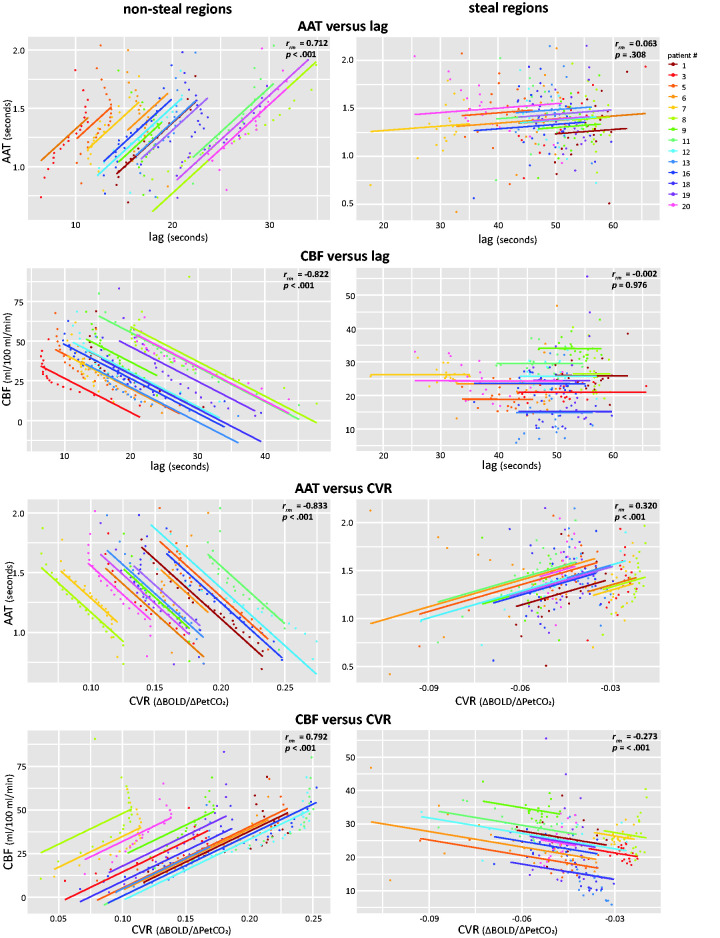
Repeated measures correlation plots between ASL metrics (AAT, CBF) and CVR metrics (CVR, hemodynamic lag) using AAT-binned or CBF-binned ROIs. Each color represents a different patient and the lines represent the common linear relationship between the MRI metrics when all participants are taken into account. Statistics for each relationship are provided for each plot separately. Correlations between ASL metrics (AAT and CBF) and CVR metrics (CVR and hemodynamic lag) were also performed and are shown in Supplementary Figure 3. AAT: arterial arrival time; CBF: cerebral blood flow; CVR: cerebrovascular reactivity; ROI: region of interest.

The data provided by steps 1–4 were used in the subsequent comparative analyses. First the previously generated masks were used to subdivide the brain into GM, WM, edema and brain metastases regions (Figure 2(A)). Next, for steal and non-steal regions separately, the mean value per brain regions was calculated for each hemodynamic parameter map. Kruskal-Walis H-tests were performed to compare the mean hemodynamic values between tissue types. To reduce the false discovery rate due to multiple testing, alpha’s were corrected according to the Benjamini-Hochberg method.^33^ Additionally, Spearman’s correlation analyses were performed between the mean values of the different parameter maps within each tissue type.

For a second comparative analysis, the AAT data was sorted based on ascending values. The sorted AAT’s were then subdivided into 20 bins, each containing 5 percent of the data. Using the boundaries of these ‘5%-bins’, the AAT map was divided into ROIs. Thereby, each ROI contained 5 percent of the AAT data, where the lowest bin value represented the 5 percent lowest AAT values and the highest bin value the 5 percent highest AAT values (Figure 2(B)). For each hemodynamic parameter, the mean value for each of these binned ROIs was calculated and used in the subsequent correlation analysis to assess whether the hemodynamic parameters in each AAT bin correlate with other hemodynamic parameters. In order to compare CBF values to the CVR metrics as well, this process was repeated using the CBF maps to bin the data. As this binning resulted in 20 mean values per individual (i.e. for each binned ROI), a repeated-measures correlation was chosen to account for the within-individual variance between these values. The ‘rmcorr’ package implemented in RStudio (Version 2021.9.1.372) was used to perform this repeated-measures correlation.^34^ This analysis estimates the common regression slope for repeated measures.

Lastly, a Pearson’s correlation coefficient was calculated for the voxelwise association between the ASL and CVR metrics for each individual. This correlation was performed separately for non-vascular steal and vascular steal regions. Next, we performed a nonparametric Wilcoxon-signed rank-test to assess whether the correlation values significantly differed from zero on the group-level (See Supplementary Results). For all statistical tests a *p*-value of 0.05 was deemed significant.

## Results

### Participants

In total, 20 patients were included in the APRICOT study in the given time period. Data of 14 of these participants was used in the current analysis and 6 patients were excluded due to various, mostly technical reasons (see Supplementary Table 1). The average age of the included patients was 65.1 years and four were female. On average patients had 5.4 brain metastases of which the average volume of the newly treated, non-resected brain metastases was 10.7 cc. Edema encompassed on average 23.93 cc of the brain. Two patients had previous resection of at least one BM and three patients had received previous brain radiotherapy. On average, 62.30 cc of the brain volume (5.7% of the total brain volume) showed steal phenomena as indicated by negative CVR values (see Table 1). On average 13.4% (range: 0–43.6%) of the brain metastases volume and in 21.9% (range: 0–52.3%) of the edema volume showed steal.

**Table 1.** Patient, treatment and volume information of the included subjects in this study.

Patient number	Age (years)	Sex	Primary tumor	Number of BMs	BMs volume (cc)	Previous resection	Previous RT	Steal volume (cc)	Edema volume (cc)
APP001	56	F	Gynecological	3	–	Y	Y	37.03	4.36
APP003	57	F	Lung	7	4.32	N	N	33.41	54.27
APP005	66	M	Melanoma	18	0.87	N	Y	57.81	12.46
APP006	81	M	Melanoma	2	7.09	N	N	40.40	17.10
APP007	62	F	Lung	11	11.20	N	N	74.75	19.46
APP008	72	M	Kidney	1	17.56	N	N	105.29	80.14
APP009	67	M	Lung	8	1.17	N	N	77.20	1.48
APP011	52	F	Lung	8	50.64	N	N	61.71	29.41
APP012	58	M	Melanoma	9	0.67	N	N	28.04	0.30
APP013	75	M	Melanoma	2	9.44	N	N	54.61	1.76
APP016	53	M	Melanoma	2	0.13	N	Y	43.71	–
APP018	72	M	Lung	2	1.75	N	N	38.70	13.19
APP019	65	M	Gastro-intestinal	1	23.85	N	N	81.66	84.43
APP020	75	M	Lung	1	–	Y	N	137.79	16.67

BMs: brain metastases; F: female; M: male; N: no; RT: radiotherapy; Y: yes.

### Mean group comparison

For each hemodynamic parameter map, the group mean values were compared between steal versus non-steal regions and between different tissue types (GM, WM, edema and brain metastases) using Kruskal-Walis tests (see Figure 3 and Supplementary Table 2). For the AAT, steal regions showed longer AAT than non-steal regions, but only in WM regions (*p* = .015). Additionally, for both non-steal and steal regions AAT values in GM were lower than in WM (non-steal: *p *= 0.022; steal: *p* = .004), edema (non-steal, *p* = .006; steal, *p* = .001) or brain metastases (non-steal, *p* = .018; steal, *p* = .007). AAT values in WM, were lower than in edema in steal regions (*p* = .031), but did not differ from edema in non-steal regions (*p *= 0.37) or brain metastases (non-steal: *p* = .111; steal *p* = .143).

Non-steal regions exhibited significantly higher CBF than steal regions in GM (*p* = .010), WM (*p* = .001) and edema (*p* = .034), but not within brain metastases (*p* = .129). Additionally, GM steal and non-steal regions showed higher CBF than WM (non-steal: *p *= .001; steal: *p < *.001) and edema (non-steal: *p < *.001; steal: *p < *.001). CBF was also higher in WM than edema, but only in the non-steal regions (non-steal: *p *= .004; steal: *p *= .136). While brain metastases showed high variability in CBF values, on average CBF values in brain metastases were higher than in WM (non-steal: *p *= .005; steal: *p *= .014) and edema (non-steal: *p < *.001; steal: *p *= .007), but did not differ from GM values (non-steal: *p *= .572; steal: *p *= .953).

Hemodynamic lag values were shorter in non-steal than steal regions in GM (*p* < .001), WM (*p* < .001) and edema regions (*p* = .002), but not within brain metastases (*p* = .056). For non-steal regions both WM and edema had longer lag times than GM (*p* = .010 and *p* < .001, respectively). Additionally, edema exhibited longer lags than WM (*p* = .003). All of the steal regions exhibited similarly long lags across the tissue types.

As steal regions were defined using the negative CVR values, all steal and non-steal CVR values significantly differed from each other (*p* < .001). In non-steal regions, GM showed a higher reactivity than WM (*p* = .002), edema (*p* < .001) and brain metastases (*p* = .009). WM also had a higher reactivity than edema (*p* < .001). Within steal regions, GM had a larger negative response than both WM (*p* = .003) and edema (*p* < .001), but not brain metastases (*p *= 0.61). Within these steal regions, WM had a more negative response than edema regions (*p* = .027).

Next, Spearman’s Rho correlations were performed to test for an association between the different MRI metrics within the tissue types. For the non-steal regions, in brain metastases only hemodynamic lag significantly negatively correlated with CVR values (*r* = −0.73, *p *= 0.009). For steal regions, longer AAT was significantly related to lower perfusion (*r* = −0.534, *p* = .048), but only within GM. See Supplementary Figure 2 for scatterplots of these associations.

### Repeated measures correlation

**Figure 5.**
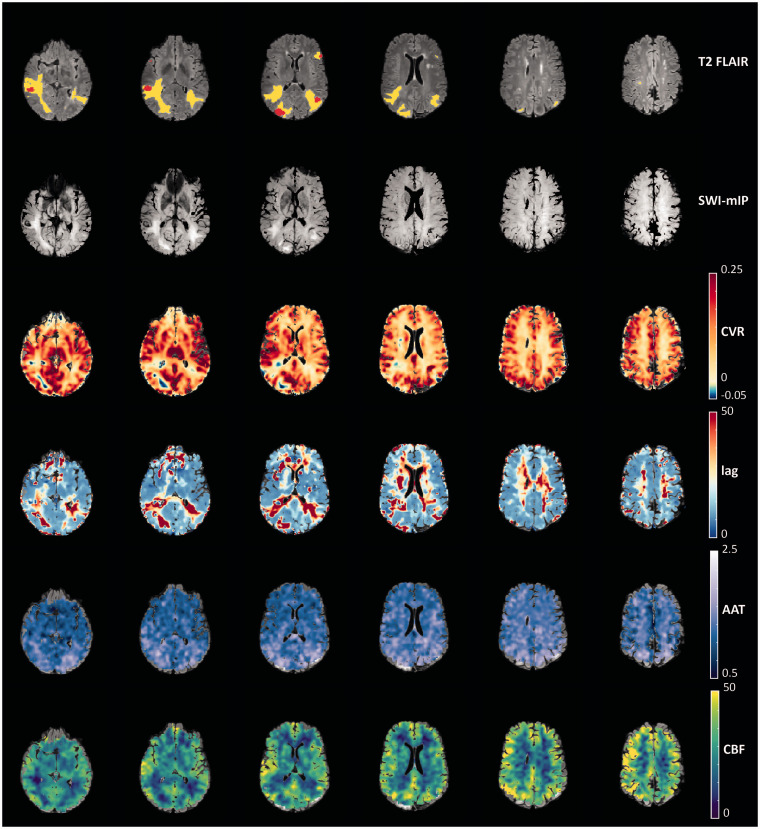
Visual comparison of hemodynamic parameter maps of a representative subject. Red voxels ona the T2 FLAIR represent the location of the brain metastases, and yellow voxels are regions with edema. The minimum intensity projection (SWI-mIP) indicates the locations of larger cerebral venous vessels. AAT: arterial arrival time; CBF: cerebral blood flow; CVR: cerebrovascular reactivity.

Repeated measures correlations were performed between each MRI metric using either the AAT binned or CBF binned ROIs (see Figure 4 and Supplementary Figure 3). In non-steal regions, longer hemodynamic lag time was associated with both longer AAT (*r_rm_ *= 0.712, *p* < .001) as well as lower CBF (*r_rm_ *= −0.822, *p* < .001). No relationship between either AAT and hemodynamic lag (*r_rm_ *= 0.063, *p* = .308) or CBF and hemodynamic lag (*r_rm_ *= −0.002, *p *= 0.976) was found for steal regions. Higher CVR was related to shorter AAT (*r_rm_ *= −0.833, *p* < .001) as well as higher CBF (*r_rm_ *= 0.792, *p* < .001) in non-steal regions. Within the steal regions this association flipped with stronger negative CVR (i.e. stronger vascular steal effect) related to shorter AAT (*r_rm_ *= 0.320, *p* < .001) and higher CBF (*r_rm_ *= −0.273, *p* < .001). Additionally, similar associations can be visually observed in the representative example patient (see Figure 5).

## Discussion

In the current study, baseline perfusion measurements were compared with functional vascular parameters acquired using CVR in a population of patients with brain metastases. These parameters were compared both spatially, and across different tissue types (GM, WM, edema and brain metastases), and also tissues exhibiting different hemodynamic characteristics (steal versus non-steal regions). When visually inspecting the MRI data, all vascularly compromised regions visible in the ASL data were also reflected in the CVR metrics. Results indicate a strong, positive relationship between regional baseline CBF and CVR. Additionally, there was a strong positive relationship between the temporal metrics AAT and hemodynamic lag. In both instances, however, these relationships do not hold in brain regions with exhausted cerebral autoregulation (i.e. steal regions). Thus, while ASL may be able to inform on some functional vascular aspects, CVR provides additional information regarding brain tissue at risk as indicated by either vascular steal or increased hemodynamic lag.

Tissue-specific hemodynamic characteristics were observed in our results. WM areas were characterized by lower CBF and longer AAT, which has been previously reported in healthy subjects.^35^ Additionally, WM areas showed lower reactivity as well as longer hemodynamic lag values than GM. This is consistent with existing literature, and reflects lower WM blood volume, different venous draining architecture and/or potential flow redistribution resulting from the strong GM CO_2_ response.^29,31,36^ Our findings suggest that the hemodynamic characteristics of healthy tissue are preserved in the normal appearing brain tissue of patients with brain metastases . On the other hand, large variability in the hemodynamic vascular parameters was seen within tissue containing untreated brain metastases. This was reflected in both the ASL and the CVR parameters. High variability in CBF measurements has previously been shown both between patients with brain metastases as well as between multiple brain metastases within patients.^37^ The primary tumor (i.e. origin of metastatic cells), might cause some of this heterogeneity as it affects the brain metastases growth pattern.^15,38,39^ Our findings additionally show that edema regions are characterized by lower CBF, lower CVR and both longer AAT and hemdoynamic lag times. Previous literature has also found impaired CVR within perifocal edema surrounding diffuse gliomas.^13^ It could be speculated that the local pressure in edematous regions restricts the ability of vessels to dilate and thus cannot maintain adequate perfusion. Additionally, this could limit the vasodilatory reserve capacity, as reflected by the CVR parameters.

GM CBF values varied between 30 and 50 ml/100 ml/min in our patient sample, which is similar to the GM CBF values as measured using PET.^40^ CBF and CVR values were strongly correlated within adequately functioning regions, both when performing ROI and voxelwise statistics. That is, regions with lower blood supply also showed lower vascular reactivity. This was to be expected since CVR measurements are heavily dependent on changes in CBF. Additionally, longer hemodynamic lag times were seen within regions with lower CBF. This has also been observed in healthy subjects and patients with white-matter hyperintensities, where reduced CBF was found alongside lower CVR and longer CVR time to peak.^41–44^ In the current study, however, in regions exhibiting vascular steal the negative CVR decreased further with increasing CBF. Even though the effect size of this relationship is considered small,^45^ this result is still striking. Previous research has likewise postulated that the relationship between CBF and CVR may be dependent on the staging of vascular reserve.^8^ This could be explained by the following: within non-steal regions (i.e. adequately responsive regions), the process of cerebral autoregulation ensures CBF is maintained through varying the vasodilation of the vessels. However, in vascular steal regions, vessels are likely to be maximally dilated. When this maximum vascular reserve capacity is able to compensate, adequate CBF could be maintained while CVR is negative. In even more vascularly comprised regions, this maximum vasodilation fails to compensate, leading to both low CBF and CVR values. These different associations could be reflective of the underlying vascular reserve clasifications as proposed by both Derdeyn and colleagues^46^ or Kuroda and colleagues^47^.

The timing of the response as measured with CVR was related to the temporal metrics of the baseline CBF (i.e. AAT). In other words, regions in which the arrival time of labeled blood was longer also showed a longer vascular response delay to a hypercapnic stimulus. Since the timing of the CVR is related to the traveling duration of the blood, this positive relationship was to be expected. However, both the correlation analyses as well as visual inspection of the data suggest these two temporal measures are not identical. Within the same region, the hemodynamic lag time is longer than the AAT. This probably reflects the difference in the two temporal metrics. AAT solely reflects the arrival time of labeled blood in a physiological steady state on the arterial side. Hemodynamic lag, on the other hand, is a time measure in reaction to a hypercapnic stimulus, measured at the venous side and influenced by multiple factors like blood redistribution and vascular response speed.^29^ While prolonged AAT might be able to indicate areas with possible increased vascular collateralization^48^ and thereby also longer hemodynamic lag values, AAT cannot be used to identify regions at risk for decreased vascular response speed.

When comparing ASL with BOLD MRI data, some technical limitations of both techniques should be considered. The main limitation of ASL is the short half-life of the endogenous tracer (∼1–2 seconds), leading to possible underestimation of perfusion in areas with long transit delays.^49^ Additionally, the specific ASL technique may influence the resulting perfusion maps. An example is that too short post-labeling delays can lead to arterial transit artefacts or less accurate CBF measures.^3,50,51^ In the current study, we used a multi post-labeling delay ASL pcASL sequence and did not perform partial volume correction leading to CBF values that are close to the ground truth as measured using PET. A disadvantage of BOLD-metrics, is that the BOLD response is dependent on multiple factors, like changes in cerebral blood volume, cerebral metabolic rate of oxygen consumption, arterial partial pressure of oxygen and baseline parameters like hematocrit, OEF, CMRO_2_, and blood volume.^52^^,^^53^ This makes it difficult to pinpoint the exact underlying mechanisms and could be viewed as a less pure measure of brain hemodynamics when compared to ASL. Nevertheless, on average the change in BOLD to changes in PetCO_2_ seem to be dominated by CBF changes.^42^^,^^54^^,^^55^ Thereby it could be speculated that the BOLD response is mainly affected by changes in CBF and provides a good comparison with ASL MRI.

Additionally, specific analyses choices could have influenced results. In areas with vascular steal, low variability in CVR values in combination with highly variable hemodynamic lag values were observed. As steal regions were defined by any CVR value below zero, these regions could thereby include voxels containing noisy, just below threshold CVR values, thereby influencing the results. Moreover, as the current analysis pipeline is influenced by the time to peak for the hemodynamic lag calculation, it may be difficult to discern the actual lag time of the response. Thereby hemodynamic lag values will not always reflect true underlying temporal characteristics of tissue reactivity. Therefore, we advise not to perform lag analysis within regions indicated by vascular steal as it will possibly lead to spurious lag quantifications.

To further understand the exact hemodynamic mechanisms, oxygen extraction fraction (OEF) would be a valuable additional metric. To illustrate, in regions with exhausted cerebral autoregulation, a further reduction in cerebral perfusion pressure will cause CBF to drop. In order to maintain tissue function, OEF can be increased. The functional consequences of reduced regional CBF will therefore only become apparent when OEF is maximal.^4^ Thus, future studies should add OEF measurements to further understand whether tissue at risk as indicated by either ASL or CVR is also reflective of tissue with maximized OEF. To fully understand the functional consequences of these hemodynamic measure, the MRI metrics should be related to behavioral measures, like cognitive performance.

In the current study, we investigated how ASL parameters measured during a physiological steady state relate to functional vascular parameters as measured using CVR in a patient population with brain metastases. When visually inspecting the MRI data, all vascularly compromised regions visible in the ASL data were also reflected in the CVR metrics. This was confirmed by both the regional and voxelwise relationship between on the one hand CBF and CVR measurements and the temporal metrics of ASL and CVR on the other hand. However, the relationship between ASL and CVR measures seems to be dependent on the vascular status of the underlying tissue. That is, relationships do not hold in tissues exhibiting vascular steal. Thus, CVR metrics may be able to flag at-risk areas before they become visible on ASL MRI. However, the downside of using BOLD-metrics is that they are influenced by multiple variables, making it difficult to pinpoint the exact mechanisms underlying this vascular risk. Consequently, to fully understand vascular changes within patients with pathology, combining ASL and CVR will provide a more complete picture.

## Supplemental Material

sj-pdf-1-jcb-10.1177_0271678X231196989 - Supplemental material for Hemodynamic imaging parameters in brain metastases patients – Agreement between multi-delay ASL and hypercapnic BOLD Supplemental material, sj-pdf-1-jcb-10.1177_0271678X231196989 for Hemodynamic imaging parameters in brain metastases patients – Agreement between multi-delay ASL and hypercapnic BOLD by Eva E van Grinsven, Jamila Guichelaar, Marielle EP Philippens, Jeroen CW Siero and Alex A Bhogal in Journal of Cerebral Blood Flow & Metabolism

## Data Availability

Upon completion of the APRICOT trial data can be made available pending a formal research proposal. Analysis scripts are openly available via the SeeVR toolbox (https://www.seevr.nl/).

## References

[1] Alsop DC Detre JA Golay X , et al. Recommended implementation of ASL perfusion MRI for clinical applications. Magn Reson Med 2015; 73: 102–116. 24715426 10.1002/mrm.25197 PMC4190138

[bibr2-0271678X231196989] Detre JA Rao H Wang DJJ , et al. Applications of arterial spin labeled MRI in the brain. J Magn Reson Imaging 2012; 35: 1026–1037. 22246782 10.1002/jmri.23581 PMC3326188

[3] Telischak NA Detre JA Zaharchuk G. Arterial spin labeling MRI: Clinical applications in the brain. J Magn Reson Imaging 2015; 41: 1165–1180. 25236477 10.1002/jmri.24751

[4] Markus HS. Cerebral perfusion and stroke. J Neurol Neurosurg Psychiatry 2004; 75: 353–361. 14966145 10.1136/jnnp.2003.025825 PMC1738983

[bibr5-0271678X231196989] Sebök M Niftrik CV Wegener S , et al. Agreement of novel hemodynamic imaging parameters for the acute and chronic stages of ischemic stroke: a matched-pair cohort study. Neurosurg Focus 2021; 51: E12. 10.3171/2021.4.FOCUS21125 34198249

[6] Václavů L Meynart BN Mutsaerts HJMM , et al. Hemodynamic provocation with acetazolamide shows impaired cerebrovascular reserve in adults with sickle cell disease. Haematologica 2019; 104: 690–699. 30523051 10.3324/haematol.2018.206094 PMC6442969

[7] Chan ST Evans KC Rosen BR , et al. A case study of magnetic resonance imaging of cerebrovascular reactivity: a powerful imaging marker for mild traumatic brain injury. Brain Inj 2015; 29: 403–407. 25384127 10.3109/02699052.2014.974209 PMC4440418

[8] Sobczyk O Battisti-Charbonney A Fierstra J , et al. A conceptual model for CO_2_-induced redistribution of cerebral blood flow with experimental confirmation using BOLD MRI. Neuroimage 2014; 92: 56–68. 24508647 10.1016/j.neuroimage.2014.01.051

[9] Champagne AA Bhogal AA Coverdale NS , et al. A novel perspective to calibrate temporal delays in cerebrovascular reactivity using hypercapnic and hyperoxic respiratory challenges. Neuroimage 2019; 187: 154–165. 29217405 10.1016/j.neuroimage.2017.11.044

[bibr10-0271678X231196989] Frederick B B Nickerson LD Tong Y. Physiological denoising of BOLD fMRI data using regressor interpolation at progressive time delays (RIPTiDe) processing of concurrent fMRI and near-infrared spectroscopy (NIRS). Neuroimage 2012; 60: 1913–1923. 22342801 10.1016/j.neuroimage.2012.01.140 PMC3593078

[11] Tong Y Bergethon PR Frederick B B. An improved method for mapping cerebrovascular reserve using concurrent fMRI and near-infrared spectroscopy with regressor interpolation at progressive time delays (RIPTiDe). Neuroimage 2011; 56: 2047–2057. 21459147 10.1016/j.neuroimage.2011.03.071 PMC3134125

[12] Cai S Shi Z Zhou S , et al. Cerebrovascular dysregulation in patients with glioma assessed with time-shifted BOLD fMRI. *Radiology* 2022; 304: 155–163. 10.1148/radiol.212192 35380491

[bibr13-0271678X231196989] Fierstra J van Niftrik C Piccirelli M , et al. Diffuse gliomas exhibit whole brain impaired cerebrovascular reactivity. Magn Reson Imaging 2018; 45: 78–83. 28986176 10.1016/j.mri.2017.09.017

[14] Sebök M van Niftrik CHB Muscas G , et al. Hypermetabolism and impaired cerebrovascular reactivity beyond the standard MRI-identified tumor border indicate diffuse glioma extended tissue infiltration. Neurooncol Adv 2021; 3: vdab048. 34056603 10.1093/noajnl/vdab048 PMC8156976

[15] Kienast Y von Baumgarten L Fuhrmann M , et al. Real-time imaging reveals the single steps of brain metastasis formation. Nat Med 2010; 16: 116–122. 20023634 10.1038/nm.2072

[16] Fidler IJ Yano S Zhang RD , et al. The seed and soil hypothesis: vascularisation and brain metastases. Lancet Oncol 2002; 3: 53–57. 11905606 10.1016/s1470-2045(01)00622-2

[bibr17-0271678X231196989] Langley RR Fidler IJ. The biology of brain metastasis. Clin Chem 2013; 59: 180–189. 23115057 10.1373/clinchem.2012.193342

[18] García-Gómez P Valiente M. Vascular co-option in brain metastasis. Angiogenesis 2020; 23: 3–8. 31701335 10.1007/s10456-019-09693-x

[19] El Kamar FG Posner JB. Brain metastases. Semin Neurol 2004; 24: 347–362. 15637647 10.1055/s-2004-861530

[20] World Medical Association. World medical association declaration of Helsinki: ethical principles for medical research involving human subjects. JAMA 2013; 310: 2191–2194. 24141714 10.1001/jama.2013.281053

[21] Jenkinson M Beckmann CF Behrens TE , et al. FSL. Neuroimage 2012; 62: 782–790. 21979382 10.1016/j.neuroimage.2011.09.015

[22] Jenkinson M Bannister P Brady M , et al. Improved optimization for the robust and accurate linear registration and motion correction of brain images. Neuroimage 2002; 17: 825–841. 12377157 10.1016/s1053-8119(02)91132-8

[23] Zhang Y Brady M Smith S. Segmentation of brain MR images through a hidden Markov random field model and the expectation-maximization algorithm. IEEE Trans Med Imaging 2001; 20: 45–57. 11293691 10.1109/42.906424

[24] Schmidt P Gaser C Arsic M , et al. An automated tool for detection of FLAIR-hyperintense white-matter lesions in multiple sclerosis. Neuroimage 2012; 59: 3774–3783. 22119648 10.1016/j.neuroimage.2011.11.032

[25] Jenkinson M Smith SM. A global optimisation method for robust affine registration of brain images. Med Image Anal 2001; 5: 143–156. 11516708 10.1016/s1361-8415(01)00036-6

[26] Andersson JLR Skare S Ashburner J. How to correct susceptibility distortions in spin-echo echo-planar images: application to diffusion tensor imaging. Neuroimage 2003; 20: 870–888. 14568458 10.1016/S1053-8119(03)00336-7

[27] Smith SM Jenkinson M Woolrich MW , et al. Advances in functional and structural MR image analysis and implementation as FSL. Neuroimage 2004; 23 Suppl 1: S208–S219. 15501092 10.1016/j.neuroimage.2004.07.051

[28] Bhogal AA. abhogal-lab/SeeVR: V2.01. Published online 2021: (V2.01). DOI: 10.5281/zenodo.6532362

[29] Champagne AA Bhogal AA. Insights into cerebral tissue-specific response to respiratory challenges at 7T: evidence for combined blood flow and CO_2_-mediated effects. Front Physiol 2021; 12: 601369. 33584344 10.3389/fphys.2021.601369 PMC7876301

[30] Donahue MJ Strother MK Lindsey KP , et al. Time delay processing of hypercapnic fMRI allows quantitative parameterization of cerebrovascular reactivity and blood flow delays. J Cereb Blood Flow Metab 2016; 36: 1767–1779. 26661192 10.1177/0271678X15608643 PMC5076782

[31] Bhogal AA. Medullary vein architecture modulates the white matter BOLD cerebrovascular reactivity signal response to CO_2_: observations from high-resolution T2 weighted imaging at 7T. Neuroimage 2021; 245: 118771. 34861395 10.1016/j.neuroimage.2021.118771

[32] Chappell MA Groves AR Whitcher B , et al. Variational Bayesian inference for a nonlinear forward model. IEEE Trans Signal Process 2009; 57: 223–236.

[33] Benjamini Y Hochberg Y. Controlling the false discovery rate : a practical and powerful approach to multiple testing author (s): Yoav Benjamini and Yosef Hochberg source : Journal of the royal statistical society. Series B (methodological), vol. 57, no. 1 (1995), publi. J R Stat Soc 1995; 57: 289–300.

[34] RStudio Team (2020). RStudio: Integrated Development for R. RStudio, PBC, Boston, MA. http://www.rstudio.com/.

[35] Van Osch MJP Teeuwisse WM Van Walderveen MAA , et al. Can arterial spin labeling detect white matter perfusion signal? Magn Reson Med 2009; 62: 165–173. 19365865 10.1002/mrm.22002

[36] Bhogal AA De Vis JB Siero JCW , et al. The BOLD cerebrovascular reactivity response to progressive hypercapnia in young and elderly. Neuroimage 2016; 139: 94–102. 27291492 10.1016/j.neuroimage.2016.06.010

[37] Lassen U Andersen P Daugaard G , et al. Brain metastases from small cell lung cancer respond to chemotherapy, but response duration is short and the intracerebral concentration of chemotherapy may be too low because of the characteristics of the blood-brain barrier. Positron emission tomography. Clin Cancer Res 1998; 4: 2591–2597. 9829721

[38] Quattrocchi CC Errante Y Mallio CA , et al. Brain metastatic volume and white matter lesions in advanced cancer patients. J Neurooncol 2013; 113: 451–458. 23666234 10.1007/s11060-013-1137-z

[39] Berk BA Nagel S Hering K , et al. White matter lesions reduce number of brain metastases in different cancers: a high-resolution MRI study. J Neurooncol 2016; 130: 203–209. 27535745 10.1007/s11060-016-2235-5

[40] Yamaguchi T Kanno I Uemura K , et al. Reduction in regional cerebral metabolic rate of oxygen during human aging. Stroke 1986; 17: 1220–1228. 3492786 10.1161/01.str.17.6.1220

[41] Leoni RF Oliveira IAF Pontes-Neto OM , et al. Cerebral blood flow and vasoreactivity in aging: an arterial spin labeling study. Braz J Med Biol Res 2017; 50: e5670. 28355354 10.1590/1414-431X20175670 PMC5423749

[bibr42-0271678X231196989] Mandell DM Han JS Poublanc J , et al. Mapping cerebrovascular reactivity using blood oxygen level-dependent MRI in patients with arterial steno-occlusive disease: comparison with arterial spin labeling MRI. Stroke 2008; 39: 2021–2028. 18451352 10.1161/STROKEAHA.107.506709

[bibr43-0271678X231196989] Lu H Xu F Rodrigue KM , et al. Alterations in cerebral metabolic rate and blood supply across the adult lifespan. Cereb Cortex 2011; 21: 1426–1434. 21051551 10.1093/cercor/bhq224 PMC3097991

[44] Marstrand JR Garde E Rostrup E , et al. Cerebral perfusion and cerebrovascular reactivity are reduced in white matter hyperintensities. Stroke 2002; 33: 972–976. 11935046 10.1161/01.str.0000012808.81667.4b

[45] Cohen J. The effect size index: d. *Statistical power analysis for the behavioral sciences*. Abingdon-on-Thames: Routledge Academic. 1988.

[46] Derdeyn CP Videen TO Yundt KD , et al. Variability of cerebral blood volume and oxygen extraction: stages of cerebral haemodynamic impairment revisited. Brain 2002; 125: 595–607. 11872616 10.1093/brain/awf047

[47] Kuroda S Kamiyama H Abe H , et al. Acetazolamide test in detecting reduced cerebral perfusion reserve and predicting long-term prognosis in patients with internal carotid artery occlusion. Neurosurgery 1993; 32: 912–919. 8327091 10.1227/00006123-199306000-00005

[48] Lou X Yu S Scalzo F , et al. Multi-delay ASL can identify leptomeningeal collateral perfusion in endovascular therapy of ischemic stroke. Oncotarget 2017; 8: 2437–2443. 27974692 10.18632/oncotarget.13898 PMC5356813

[49] Wang DJJ Alger JR Qiao JX , UCLA Stroke Investigators et al. The value of arterial spin-labeled perfusion imaging in acute ischemic stroke: Comparison with dynamic susceptibility contrast-enhanced MRI. Stroke 2012; 43: 1018–1024. 22328551 10.1161/STROKEAHA.111.631929 PMC3314714

[50] Alsop DC Detre JA Golay X , et al. Recommended implementation of arterial spin labeled perfusion MRI for clinical applications: a consensus of the ISMRM perfusion study group and the European consortium for ASL in dementia. Magn Reson Med 2015; 73: 102–116. 24715426 10.1002/mrm.25197 PMC4190138

[51] Fan AP Guo J Khalighi MM , et al. Long-delay arterial spin labeling provides more accurate cerebral blood flow measurements in moyamoya patients: a simultaneous positron emission tomography/MRI study. Stroke 2017; 48: 2441–2449. 28765286 10.1161/STROKEAHA.117.017773 PMC8006795

[52] Hoge RD Atkinson J Gill B , et al. Investigation of BOLD signal dependence on CBF and CMRO_2_: the deoxyhemoglobin dilution model. Neuroimage 1999; 9: 849–863. 10.1002/(sici)1522-2594(199911)42:5<849::aid-mrm4>3.0.co;2-z 10542343

[53] Petersen ET Zimine I Ho YCL , et al. Non-invasive measurement of perfusion: a critical review of arterial spin labelling techniques. BJR 2006; 79: 688–701. 16861326 10.1259/bjr/67705974

[54] Shiino A Morita Y Tsuji A , et al. Estimation of cerebral perfusion reserve by blood oxygenation level-dependent imaging: comparison with single-photon emission computed tomography. J Cereb Blood Flow Metab 2003; 23: 121–135. 12500097 10.1097/01.WCB.0000037546.46809.CA

[55] Ziyeh S Rick J Reinhard M , et al. Blood oxygen level-dependent MRI of cerebral CO_2_ reactivity in severe carotid stenosis and occlusion. Stroke 2005; 36: 751–756. 15705935 10.1161/01.STR.0000157593.03470.3d

