# Supplementary material for: Hemodynamic imaging parameters in brain metastases patients – Agreement between multi-delay ASL and hypercapnic BOLD
Source: J Cereb Blood Flow Metab. 2023 Aug 26;43(12):2072–84. doi: 10.1177/0271678X231196989 (PMC10925872; doi:10.1177/0271678X231196989)
Supplement: sj-pdf-1-jcb-10.1177_0271678X231196989 - Supplemental material for Hemodynamic imaging parameters in brain metastases patients – Agreement between multi-delay ASL and hypercapnic BOLD [file sj-pdf-1-jcb-10.1177_0271678X231196989.pdf]

Supplementary materials

Supplementary Methods: In – and exclusion criteria

Inclusion criteria:

- Age ≥ 18 years;
- Either radiographic and/or histologic proof of metastatic brain disease eligible for brain radiation therapy;
- Eligible for brain irradiation for prophylaxis or treatment;
- Expected survival ≥ 5 months, as determined by Graded Prognostic Assessment (GPA) score;
- Sufficient knowledge of the Dutch language to allow reliable use of the standardized tests and understand the study information;
- Participation in the COIMBRA cohort, with given consent for filling in quality of life questionnaires.

Exclusion criteria:

- Standard contraindications for 3T MRI scanning;
- Standard contraindications for using the RespirAct RA-MRTM MRI UNIT (see D2 IMDD 2.5 contraindications);
- Medical contraindications to limited hypercapnia (known metabolic acidosis or alkalosis);
- Unwilling or unable to cooperate with breathing manoeuvres or keeping still;
- Noncompliance with prescribed anti-seizure medication;
- Severe current neurological or psychiatric diseases not related to the primary malignancy or cerebral metastases;
- History of cerebrovascular disease (ischaemic stroke or intracranial haemorrhage);
- Non-prophylactic use of > 4 mg dexamethasone on the day of participation;
- Cardiovascular disease: congestive heart failure (New York Heart Association Class III to IV), symptomatic ischemia, conduction abnormalities uncontrolled by conventional intervention, and myocardial infarction within past 6 months;
- Pulmonary disease: oxygen dependency at rest or with exercise, restrictive lung disease with resting respiratory rate over 15 breaths/min;
- Concurrent severe or uncontrolled medical disease (e.g., active systemic infection);
- History of bleomycin treatment;
- Body weight <30 kg or >100 kg
- Pregnancy

Supplementary Table 1

Reasons for patient exclusion for the current analysis.

Patient code	Reason for exclusion
APP002	Large artefact in parietal lobe in the BOLD time series
APP004	Patients did not complete the breathing challenges, thus no BOLD measurements were available.
APP010	Artefact in BOLD time series.
APP014	Impossible to analyze data without making individual changes to the BOLD seeVR analysis.
APP015	Breathing trace was noisy, possibly due to a leak in the mask.
APP017	No BOLD MRI scan available due to technical problems.

Abbreviations: BOLD, blood oxygenation level-dependent.

Supplementary Table 2

Group mean values (standard deviation) per MRI metric (AAT, CBF, CVR and hemodynamic lag) per tissue type (GM, WM, edema and brain metastases) divided into non-steal and steal regions.

	GM		WM		Edema		Brain metastases	
	non-steal	steal	non-steal	steal	non-steal	steal	non-steal	steal
AAT	1.27 (0.09)	1.30 (0.11)	1.35 (0.07)	1.42 (0.06)	1.42 (0.12)	1.48 (0.09)	1.46 (0.20)	1.52 (0.19)
CBF	40.36 (7.37)	32.71 (5.81)	29.61 (6.21)	20.13 (5.13)	21.81 (5.22)	17.74 (6.97)	50.33 (26.70)	39.63 (28.28)
CVR	0.19 (0.05)	-0.06 (0.02)	0.13 (0.03)	-0.04 (0.01)	0.07 (0.02)	-0.03 (0.02)	0.12 (0.07)	-0.04 (0.03)
lag	15.98 (4.60)	50.61 (6.29)	21.73 (6.09)	53.93 (7.65)	35.08 (10.87)	51.91 (10.90)	27.81 (18.20)	43.75 (17.27)

Abbreviations: AAT, arterial arrival time; CBF, cerebral blood flow*; CVR, cerebrovascular reactivity; GM, grey matter; WM, white matter.*

Supplementary Figure 1

Pre-processing step for all types of imaging data resulting in all imaging data processed and registered to BOLD space.

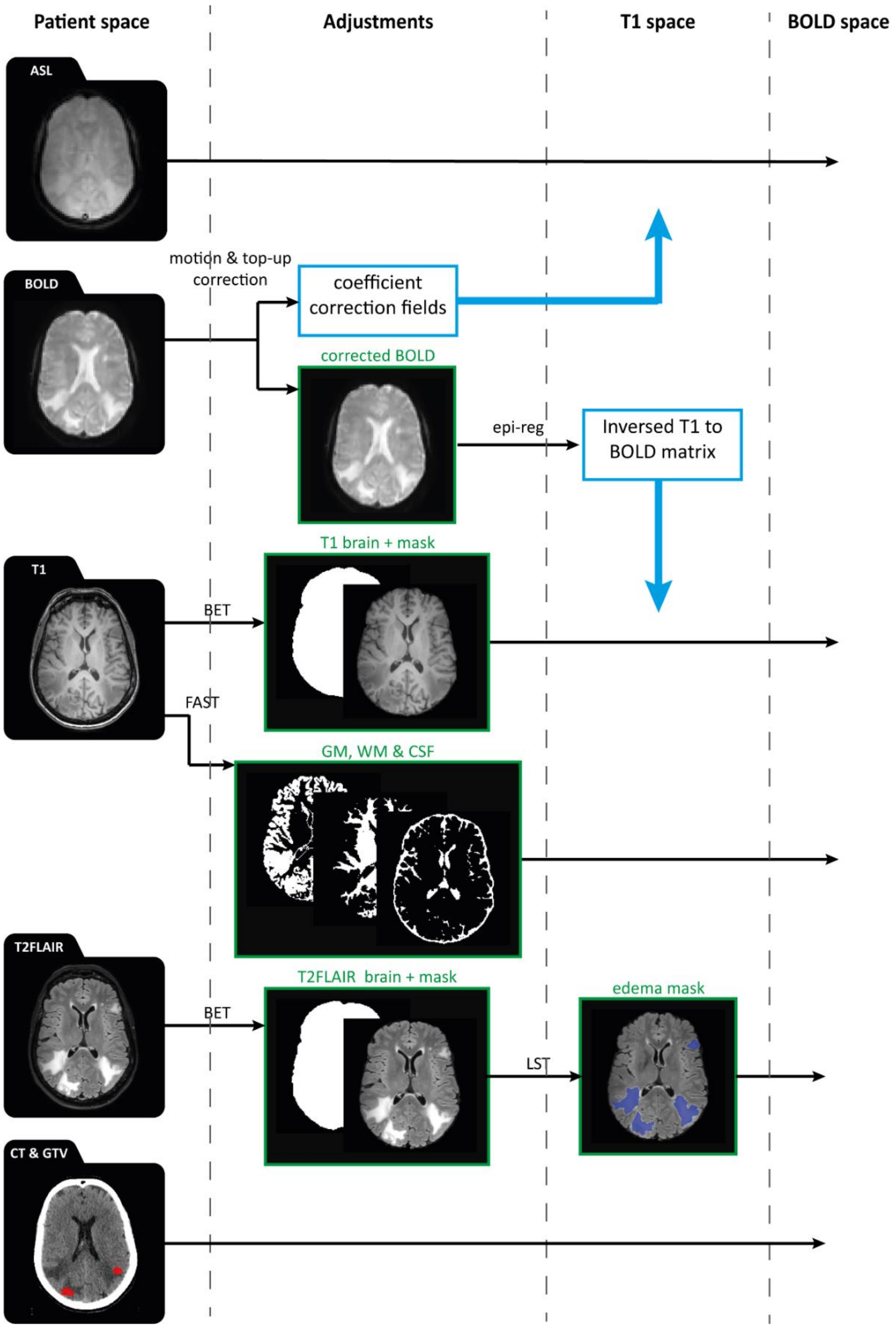

Abbreviations: ASL, arterial spin labeling; BET, Brain Extraction Tool; BOLD, blood oxygenation level-dependent; CSF, cerebrospinal fluid; FAST, FSL Automated Segmentation Tool; GM, grey matter; GTV, gross tumor volume; LST, Lesion Segmentation Tool; WM, white matter;

Supplementary Figure 2

Scatter plots for significant associations between the mean MRI metrics per tissue type divided into non-steal and steal regions.

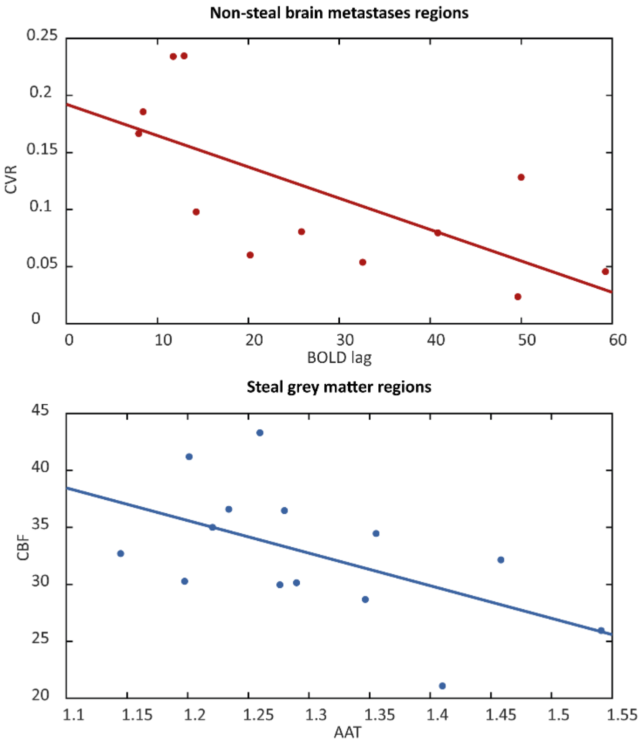

Supplementary Figure 3

Repeated measures correlation plots comparing both ASL metrics to the BOLD metrics (CVR and hemodynamic lag). Each color represents a different patient and the lines represent the linear relationship between the MRI metrics. Statistics for each relationship are provided for each plot separately.

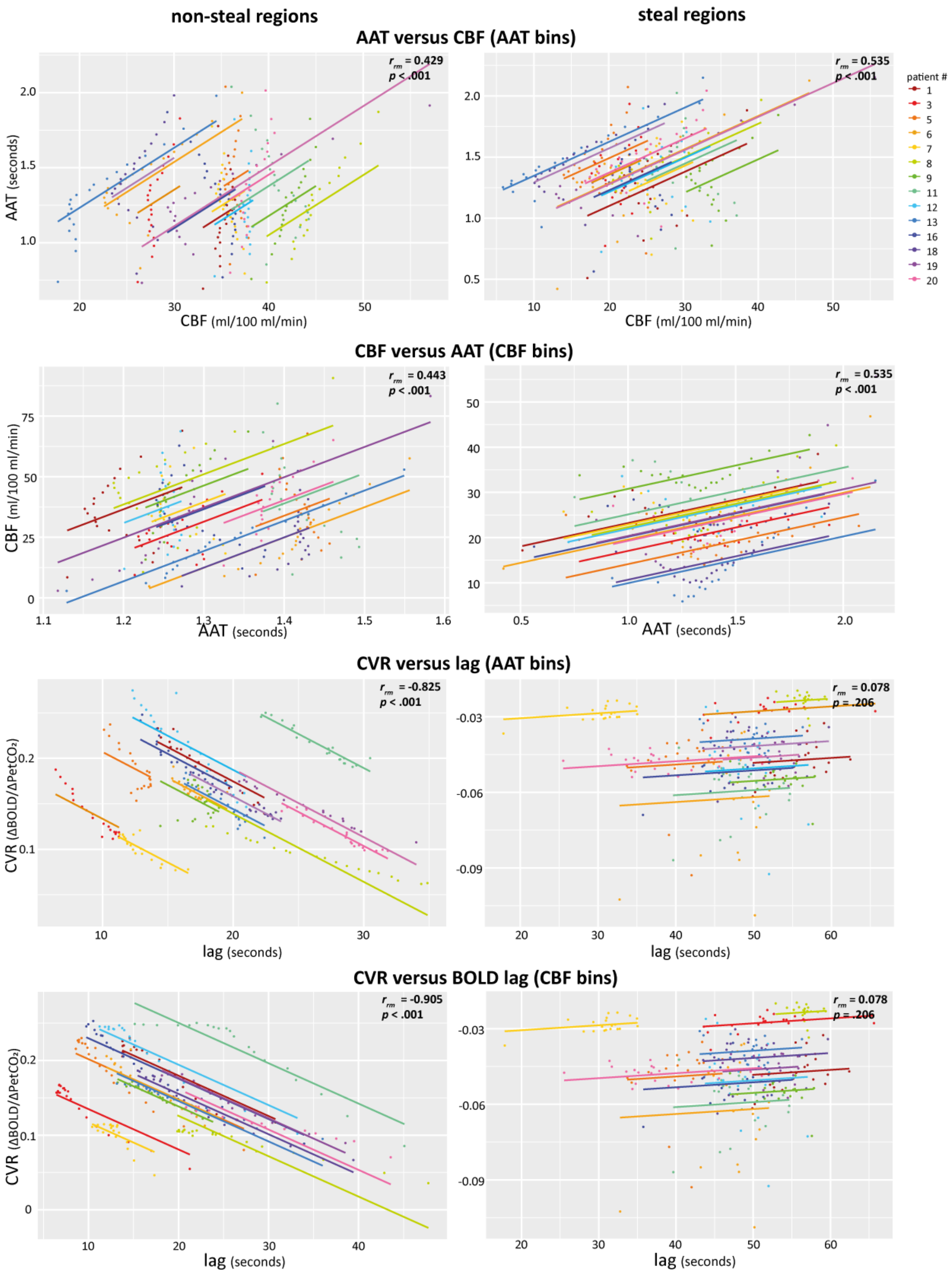

Abbreviations: AAT, arterial arrival time; ASL, arterial spin labeling; BOLD, blood oxygenation level-dependent; CBF, cerebral blood flow; CVR, cerebrovascular reactivity.

Supplementary Results

Supplementary Figure 4. Boxplots of the r-value of the voxelwise correlations between AAT, CBF, CVR and lag of all participants. Asterisks indicate correlations where the average r-value of the group was statistically different from zero.

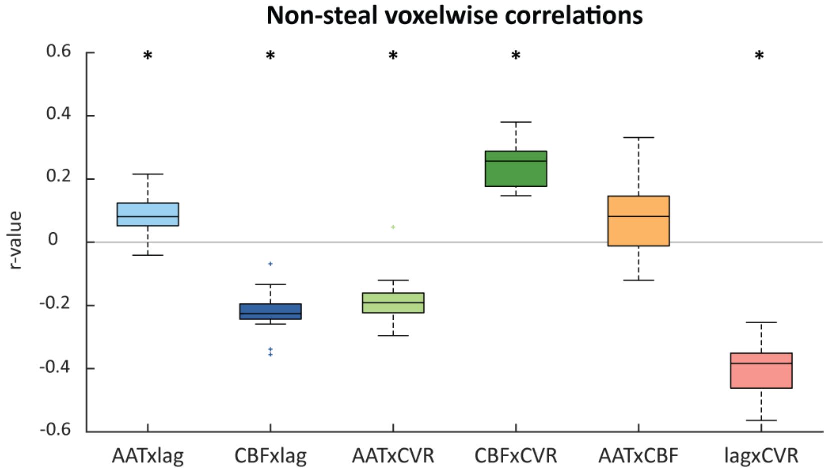

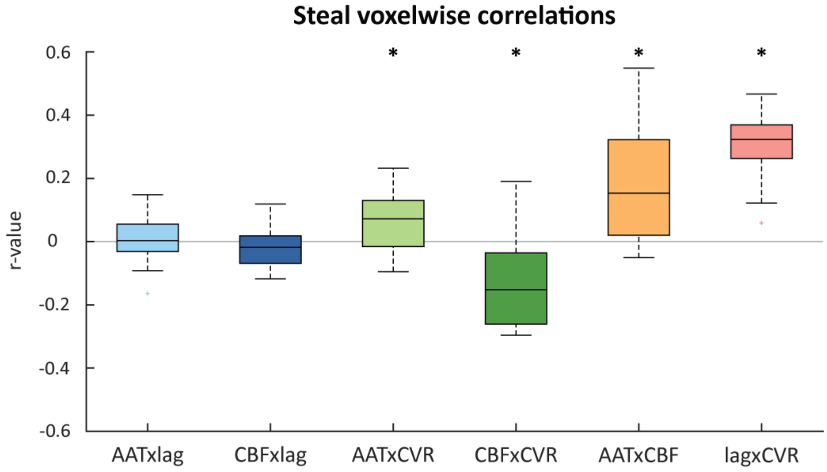

Abbreviations: AAT, arterial arrival time; CBF, cerebral blood flow; CVR, cerebrovascular reactivity.

Supplementary Table 3. P-values for the Wilcoxon signed-rank test on the correlation values for the ASL and BOLD metrics divided into areas with and without steal. Bold values indicate statistically significant results.

	Non-steal	Steal
AAT x lag	**< .001**	0.742
CBF x lag	**< .001**	0.542
AAT x CVR	**< .001**	**0.035**
CBF x CVR	**< .001**	**0.007**
AAT x CBF	0.049	**0.001**
Lag x CVR	**< .001**	**< .001**

Abbreviations: AAT, arterial arrival time; ASL, arterial spin labeling; BOLD, blood oxygenation level-dependent; CBF, cerebral blood flow; CVR, cerebrovascular reactivity.
